# Time to dig deep into the plant proteome: a hunt for low-abundance proteins

**DOI:** 10.3389/fpls.2015.00022

**Published:** 2015-01-30

**Authors:** Ravi Gupta, Yiming Wang, Ganesh K. Agrawal, Randeep Rakwal, Ick H. Jo, Kyong H. Bang, Sun T. Kim

**Affiliations:** ^1^Plant Functional Genomics Laboratory, Department of Plant Bioscience, Life and Industry Convergence Research Institute, Pusan National UniversityMiryang, South Korea; ^2^Plant Proteomics Group, Max Planck Institute for Plant Breeding ResearchCologne, Germany; ^3^Research Laboratory for Biotechnology and BiochemistryKathmandu, Nepal; ^4^Global Research Arch for Developing Education (GRADE) Academy Pvt. LtdBirgunj, Nepal; ^5^Organization for Educational Initiatives, University of TsukubaTsukuba, Japan; ^6^Department of Anatomy I, Showa University School of MedicineTokyo, Japan; ^7^Department of Herbal Crop Research, Rural Development AdministrationEumseong, South Korea

**Keywords:** low-abundance proteins, high-abundance proteins, two-dimensional gel electrophoresis, RuBisCO, post-translational modifications

## Introduction

Two-dimensional gel electrophoresis (2-DGE) has come a long way since its introduction around 40 years by the pioneering work of these three researchers (Klose, 1975; O'Farrell, 1975; Scheele, 1975). 2-DGE was one of the major breakthroughs in proteomics, enabling researchers to detect, analyze and identify the whole set of proteins of a cell or tissue, simultaneously. With the advancement in technology, some modifications to this technique like development of immobilized pH gradient (IPG) strips were introduced, which undoubtedly, made this technique more simple, rapid and autonomous (Bjellqvist et al., 1982). After its introduction to the present, 2-DGE has been the method of choice for analyzing the complex proteomes of plants. 2-DGE has been used extensively to investigate the effects of biotic and abiotic stress, role of hormones, and developmental changes of plants, among others (Agrawal and Rakwal, 2008). However, it was slowly realized that identification of the plant proteins led to the repeated detection of high-abundance proteins (HAPs) including ribulose-1,5-bisphosphate carboxylase/oxygenase (RuBisCO) and other housekeeping proteins, which are present at 10^6^-10^5^ order of magnitude (Gygi et al., 2000; Patterson and Aebersold, 2003; Görg et al., 2004). Signaling and other regulatory proteins are generally present 100 molecules per cells. Subsequently, these proteins are difficult to identify by either gel-based or gel-free proteomic approaches, even with access to the latest mass spectrometers. RuBisCO comprises of a large percentage in the total proteins and thus hinders the absorption of low-abundance proteins (LAPs) on the IPG strips, which subsequently results in the poor detection and identification of LAPs on 2D gels and by mass spectrometry (MS), respectively. Therefore, the time has come for all plant proteomers to realize the need to hunt for the LAPs, moving one step ahead from the present. As RuBisCO is the major HAP in plant leaves, here we recommend the incorporation of a RuBisCO depletion/removal method in every plant protein extraction step to look deeper into the plant proteome. RuBisCO depletion will definitely improve the proteome coverage and will lead to the detection of novel unidentified LAPs.

## Overview of the methods developed for enrichment of LAPs: merits and de-merits

### RuBisCO depletion methods

**Figure 1:**
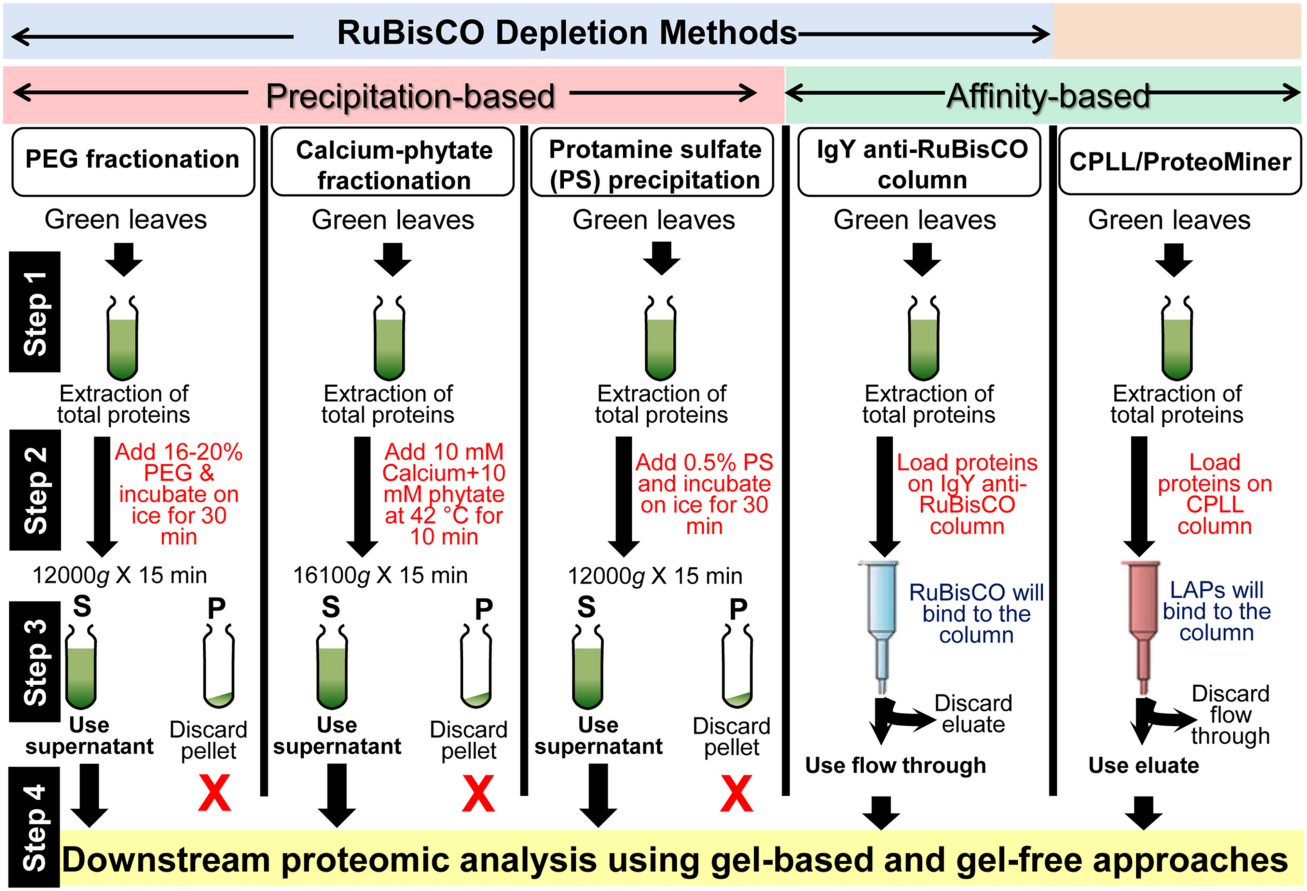
**A summary of the workflows for the enrichment of LAPs in plants using RuBisCO depletion and CPLL methods**. Details of these techniques are mentioned in the text and cited references.

A number of protein extraction methods with numerous depletion steps were developed in the last decade to remove RuBisCO, which accounts for nearly half of the total leaf protein content (summarized in Figure 1). Kim and co-workers initially developed a poly-ethylene glycol (PEG)-based method for the depletion of RuBisCO from rice leaves (Kim et al., 2001). Application of 20% PEG significantly precipitated the RuBisCO protein (large and small subunits) in the pellet fraction, resulting in the enrichment of LAPs in the supernatant fraction. Later, it was shown that 16% PEG was sufficient to deplete the RuBisCO from Arabidopsis leaves (Xi et al., 2006) indicating this method can be applied to all plants for the removal of RuBisCO. However, this PEG method is laborious and time consuming. Following the PEG fractionation approach, a new method using calcium and phytate was introduced for the removal of RuBisCO from leaves of soybean. Results revealed that a 10 min incubation of the leaf extract with 10 mM calcium and 10 mM phytate at 42°C, depleted 86% of the RuBisCO protein in the pellet fraction (Krishnan and Natarajan, 2009). As incubation of the protein extract at 42°C is absolutely essential for significant depletion of RuBisCO, this temperature condition can lead to the denaturation of some heat labile proteins. Incubation at lower temperatures significantly reduces the RuBisCO precipitation ability of this method. For example, only 44% RuBisCO depletion was achieved at 4°C (Krishnan and Natarajan, 2009). More recently, a protamine sulfate-based specific RuBisCO depletion method was introduced (Kim et al., 2013). It was shown that addition of 0.1% protamine sulfate differentially precipitates the RuBisCO in the pellet fraction and enriches the LAPs in the supernatant fraction. Using Western blotting, no RuBisCO was detected in the supernatant fraction, suggesting this method is able to deplete RuBisCO below the detection limit. 2-DGE analysis showed that application of this method in soybean resulted in visualization of 423 new spots in the supernatant fraction which were not discernible in the total fraction. Furthermore, in addition to soybean, this method was also applicable to another dicot Arabidopsis, and monocots rice and maize, suggesting that it can be universally applied in plants for the removal of RuBisCO. This protamine sulfate-based method is rapid, reliable, cost effective, and highly efficient and is more specific than the previously published PEG and calcium-phytate based methods (Kim et al., 2013).

### Immuno-affinity based methods

Other than the above mentioned precipitation methods, an affinity-based method has been developed for RuBisCO depletion. This method utilizes the anti-RuBisCO antibodies, which are commercially available and supplied as columns (IgY RuBisCO column, Sigma Aldrich; Cellar et al., 2008). The beauty of this method is that it is highly specific to RuBisCO. However, this method is very expensive limiting its wide acceptance among the scientific community, especially laboratories in the developing countries.

In addition to the RuBisCO-depletion methods, other techniques have also been introduced for the enrichment of LAPs. “Combinatorial peptide ligand library” (CPLL) technology, developed over the years by the group of P.G. Righetti, is commercially available under the trade name of Proteominer (BioRad) (Boschetti and Righetti, 2013). Briefly, CPLLs consists of several million hexapeptides (prepared using 16 different amino acids) that are able to recognize complementary amino acid sequence in a bait protein harvesting it from the sample matrix. To put is simply, when the protein extract is loaded onto a CPLL column under large overloading conditions, beads having affinity with the abundant proteins saturate first and therefore, the major fractions of these proteins are washed out due to limited binding capacity of the beads. However, due to low concentrations of LAPs, these proteins keep on binding with their partner beads when additional protein extract is loaded to it and thus get enriched (Boschetti and Righetti, 2013). This method is not based on the specific removal of RuBisCO and removes all the general HAPs from the plant extract.

## RuBisCO depletion and post-translational modification analysis

In addition to the detection of LAPs under normal conditions, depletion of RuBisCO can also be fruitful for post-translational modification (PTM) analysis. RuBisCO being phosphorylated and nitrosylated, hinders the detection of PTMs of LAPs. Recently, it was shown that removal of RuBisCO from *Brassica juncea* leaves significantly improves the detection of novel nitrosylated LAPs (Sehrawat et al., 2013). Similarly, the plant phosphoproteome coverage can also be increased by incorporating the RuBisCO depletion step during the protein extraction step, as indicated in soybean. Application of calcium phytate during protein extraction in soybean led to the identification of 28 new phosphorylated proteins which were previously undetectable, suggesting the application of RuBisCO depletion methods in PTMs discovery as well (Krishnan and Natarajan, 2009).

## Conclusions

The methods (precipitation- and affinity-based) discussed here can deplete the RuBisCO protein, a major HAP in plants. These methods are schematically presented in Figure 1. We recommend the incorporation of RuBisCO depletion step during the sample preparation for proteomics analyses given the fact that RuBisCO depletion enriches the LAPs/rare proteins. Identification of these proteins will enrich our knowledge on plant biology.

### Conflict of interest statement

The authors declare that the research was conducted in the absence of any commercial or financial relationships that could be construed as a potential conflict of interest.
